# Playing catch-up with *Escherichia coli*: using yeast to increase success rates in recombinant protein production experiments

**DOI:** 10.3389/fmicb.2014.00085

**Published:** 2014-03-05

**Authors:** Roslyn M. Bill

**Affiliations:** School of Life and Health Sciences, Aston UniversityBirmingham, UK

**Keywords:** yeast, *Saccharomyces cerevisiae*, *Pichia pastoris*, recombinant protein, yield optimization, choice of expression host

## Abstract

Several host systems are available for the production of recombinant proteins, ranging from *Escherichia coli* to mammalian cell-lines. This article highlights the benefits of using yeast, especially for more challenging targets such as membrane proteins. On account of the wide range of molecular, genetic, and microbiological tools available, use of the well-studied model organism, *Saccharomyces cerevisiae*, provides many opportunities to optimize the functional yields of a target protein. Despite this wealth of resources, it is surprisingly under-used. In contrast, *Pichia pastoris*, a relative new-comer as a host organism, is already becoming a popular choice, particularly because of the ease with which high biomass (and hence recombinant protein) yields can be achieved. In the last few years, advances have been made in understanding how a yeast cell responds to the stress of producing a recombinant protein and how this information can be used to identify improved host strains in order to increase functional yields. Given these advantages, and their industrial importance in the production of biopharmaceuticals, I argue that *S. cerevisiae* and *P. pastoris* should be considered at an early stage in any serious strategy to produce proteins.

## RECOMBINANT PROTEIN PRODUCTION IN MICROBES: *Escherichia* COLI AS THE MOST POPULAR HOST

Proteins are essential components of living organisms and have a role in virtually every cellular process: they are enzymes; form cellular scaffolds and are central to signaling, transport, and regulatory functions. To study these diverse roles, it is necessary to be able to work with sufficient quantities (typically multi-milligram) of suitably stable and functional protein samples. While some proteins can be isolated from native sources for this purpose, many cannot because they are either intrinsically unstable or are present in impractically low quantities (Bill et al., 2011). Moreover, the study of mutant or truncated forms of a given protein is often central to understanding its structure and activity; such mutants must be synthesized recombinantly.

The biotechnological breakthrough required for recombinant gene expression was first demonstrated 40 years ago in the prokaryotic microbe, *Escherichia coli* (Cohen et al., 1973) and was soon followed by the recombinant production of human somatostatin (Itakura et al., 1977) and human insulin (Goeddel et al., 1979) in *E. coli* cultures. These innovations heralded the era of the recombinant biopharmaceutical: Humulin^®^ synthesized in *E. coli* was launched by Eli Lilly and Company in 1982 (Altman, 1982); in 1987, Novo Nordisk started the industrial production of recombinant human insulin, Novolin^®^, using cultures of the eukaryotic microbe, *Saccharomyces cerevisiae* (Thim et al., 1986). Today, the recombinant production of biopharmaceuticals, particularly recombinant antibodies and vaccines, is a multi-billion dollar global business (Goodman, 2009), with more than 150 having been approved by the United States Food and Drug Administration to date (Ferrer-Miralles et al., 2009; Zhu, 2012). Approximately 20% of these biopharmaceutical proteins are produced in yeasts (the vast majority in *S. cerevisiae*), 30% in *E. coli* and 50% in mammalian cell-lines and hybridomas (Ferrer-Miralles et al., 2009; Mattanovich et al., 2012).

Research into the science of recombinant protein production is also thriving, both as an academic discipline in its own right and as a means to produce a myriad of proteins for further study (Lee et al., 2012). In 2010, it was reported that the proportion of recombinant genes expressed in *E. coli*, compared with those expressed in all hosts had remained constant, at roughly 60% per year during the 15 year period 1995–2009 (Sørensen, 2010). **Table 1** includes the corresponding data for the other commonly used host cells; it shows that the proportion of recombinant genes expressed in *E. coli* has remained high to date and that approximately half of these genes are eukaryotic. For all other hosts, the absolute numbers are much smaller, but it is notable that the proportion of recombinant genes expressed in *Pichia pastoris* has steadily increased from 1995 to date, in contrast to all other host cells (**Table 1**). Coupled with the beginnings of a decline in usage for *E. coli* over the last 8 years, this could suggest that researchers are beginning to recognize the capacity of *P. pastoris* to produce more challenging recombinant targets.

**Table 1:** Recombinant gene expression in the most commonly used host cells.

Year	All host cells	*E. coli*	*S. cerevisiae*	*P. pastoris*	Insect cells	Mammalian cell-lines
1980	0	0	0	0	0	0
1985	0	0	0	0	0	0
1990	12	75% (9; 4E)	8% (1)	0	17% (2)	0
1995	37	70% (26; 17E)	5% (2)	5% (2)	5% (2)	8% (3)
2000	50	70% (35; 17E)	0	4% (2)	12% (6)	12% (6)
2005	121	85% (103; 53E)	0	5% (6)	6% (7)	2% (2)
2010	172	76% (131; 67E)	0	9% (15)	5% (6)	5% (9)
2013	128	73% (94; 54E)	2% (2)	11% (14)	4% (5)	4% (5)

*The proportion of recombinant genes expressed in E. coli, *S. cerevisiae*, *P. pastoris*, insect cells, and mammalian cell-lines was calculated according to Sørensen’s (2010) methodology; briefly, the PubMed Central database was searched for entries containing “expression purification” in the title field, which returned 1,847 articles. These articles were categorized by year of publication and expression host used and were then examined manually to confirm the categorization. The table shows the percentage of articles reporting recombinant gene expression in a given host cell and year with the actual number in parentheses; for proteins produced in E. coli the number of recombinant proteins of eukaryotic origin (E; ranging from unicellular protozoan to human proteins) is also noted. For all other hosts, the target proteins are exclusively eukaryotic. When percentages do not total 100% in a given year, less frequently used hosts (e.g., cell-free systems and other microbes) account for the remainder.*

*Escherichia coli* stands out as the pre-eminent host cell for producing recombinant proteins in both commercial [50% of proteins; (Ferrer-Miralles et al., 2009; Mattanovich et al., 2012)] and research (>70% of proteins; **Table 1**) laboratories; it is quick and inexpensive to culture, making it ideal in many respects. However, it has been established that producing eukaryotic proteins in a prokaryotic host cell often results in inclusion body formation and/or low specific yields (Sørensen, 2010), which may be one reason for the slight decline in its more recent use (**Table 1**). An explanation for lower success rates with eukaryotic targets is that the rates of protein synthesis and folding are almost an order of magnitude faster in prokaryotes than they are in eukaryotes (Widmann and Christen, 2000). Furthermore, eukaryotic codons are often inefficiently expressed and authentic eukaryotic post-translational modifications cannot yet be achieved in *E. coli* (Sørensen, 2010). However, recent progress has been made in engineering defined glycosylation pathways in *E. coli* (Valderrama-Rincon et al., 2012), while the Keio collection of single-gene knockout mutants offers a route to understanding the molecular bottlenecks to high yields in this prokaryotic host (Baba et al., 2006).

In principle, the use of mammalian cell-lines should overcome the challenges of producing recombinant eukaryotic proteins in *E. coli*, especially with recent advances in stable recombinant gene expression (Bandaranayake and Almo, 2013; Kunert and Casanova, 2013). Furthermore, the authenticity of glycosylation performed by mammalian host cells is an important advantage over all other expression hosts. However, progress in the technologies that enable reproducible gene delivery and selection of stable clones continues to be slow (Bandaranayake and Almo, 2013). Moreover, specific yields from mammalian cell-lines are often low (Zhu, 2012) and **Table 1** shows a declining trend in their use.

Eukaryotic microbes offer substantial advantages as host cells, despite their propensity to hyperglycosylate recombinant proteins. For example, an annotated genome sequence has been available for *S. cerevisiae* for almost two decades (Goffeau et al., 1996), an impressive range of deletion and over-expression strains are readily available for *S. cerevisiae* and the *P. pastoris* genome has been available since 2009 (De Schutter et al., 2009). Combining this wealth of molecular and genetic resources, with the fact that yeasts grow an order of magnitude more rapidly than mammalian cell-lines means that protein production and optimization can be done quickly and efficiently in yeast (Porro et al., 2011). **Table 1** shows that for *P. pastoris*, at least, there is an increasing trend in its usage suggesting that these advantages have become more widely known. This is especially notable because *P. pastoris* is a relative new-comer, only having been first developed as a host system in 1985 (Cregg et al., 1985). Less elaborate hyperglycosylation, the availability of strains with humanized glycosylation pathways (Hamilton et al., 2003, 2006) and an increasing repertoire of molecular tools (Prielhofer et al., 2013) make this yeast an excellent alternative to* S. cerevisiae.* In particular, *P. pastoris* has been used with great success to produce challenging targets such as recombinant human G protein-coupled receptors and ion channels (Hedfalk, 2013); in total 19 high resolution structures have been resolved of recombinant eukaryotic membrane proteins produced in *P. pastoris *(Hedfalk, 2013). **Table 1** shows that the number of recombinant proteins produced in *S. cerevisiae* is much smaller, despite the fact that this yeast species is an important industrial host for the production of biopharmaceuticals such as hormones (e.g., insulin and human growth hormone), vaccines (against e.g., hepatitis B and human papilloma viruses), and therapeutic adjuncts (human serum albumin) (Martinez et al., 2012); this may be a consequence of the search criteria used in generating **Table 1** or possibly a perception that *S. cerevisiae* is not as amenable a host cell as *P. pastoris*.

## USING YEASTS TO INCREASE SUCCESS RATES IN RECOMBINANT PROTEIN PRODUCTION EXPERIMENTS

There is no universally applicable solution for the production of all recombinant proteins (Bill, 2001; Sørensen, 2010) and it is not yet possible to predict which host system is most likely to produce a given protein in high functional yields. To be effective, any protein production strategy should therefore encompass more than one host system.

Two main approaches are typically taken to design a new protein production experiment, preferably in combination with each other: (i) optimizing the corresponding gene sequence so it is more likely to be stably expressed and (ii) minimizing the metabolic burden on the chosen host cell(s) during recombinant protein production (Bonander and Bill, 2012). The first strategy may require that a mutant protein is produced; in support of this protein engineering approach there is an extensive literature on engineering stabilized proteins (Traxlmayr and Obinger, 2012; Scott et al., 2013). Codon optimization is also possible (Oberg et al., 2011) with more recent insights suggesting how this might aid functional expression (Halliday and Mallucci, 2014). In contrast, focusing on the host cell provides an opportunity to optimize the production of the native sequence; the principles of this second approach are broadly similar for all host cells, often requiring straightforward experimentation in the initial stages, such as optimizing culture conditions and induction protocols. Successful bioprocess engineering strategies such as these have been demonstrated to increase recombinant protein yields in cultures of both *P. pastoris* (Rebnegger et al., 2013; Spadiut et al., 2013) and *E. coli *(Jazini and Herwig, 2013). When a “Design of Experiments” (Bora et al., 2012) approach is used in this context, the effect of multiple parameters on the functional yield of recombinant protein can be examined simultaneously (Holmes et al., 2009); this is important since each input parameter is unlikely to exert an independent effect on functional protein yield (Bora et al., 2012). Successful implementation of such an approach in yeast has been shown to increase the productivity per cell by matching the methanol feed profile to the cellular metabolism (Holmes et al., 2009). In another approach, pulsing *P. pastoris* cells with methanol revealed the potential benefit of stress in increasing productivity (Dietzsch et al., 2011).

**FIGURE 1:**
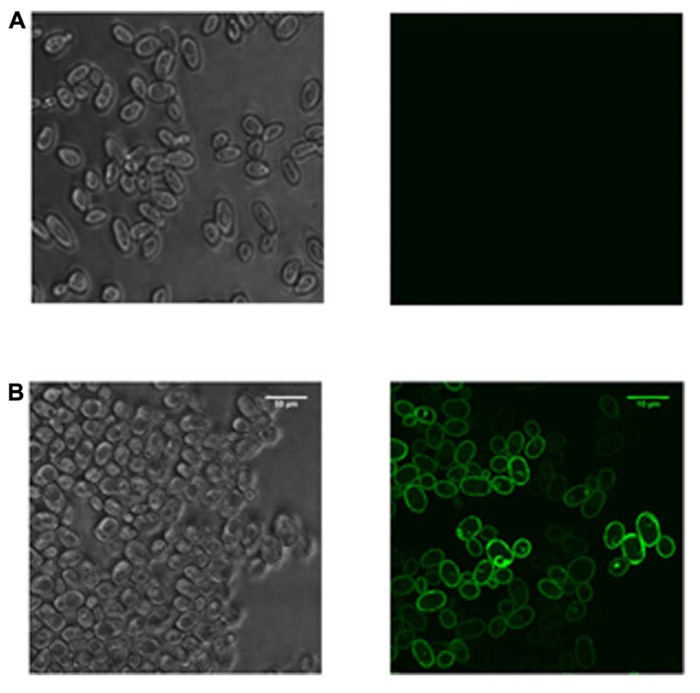
**Strain selection enables the production of a human membrane protein in *S.* cerevisiae.** Yeast cells were transformed with a plasmid expressing a construct encoding a human membrane protein tagged with green fluorescent protein. Expression was driven from a constitutive promoter and cells were imaged using confocal microscopy with an upright Leica TCS SP5 system. The sample was excited with a visible argon laser at 488 nm and imaged using a 63× oil objective. The panels show confocal images with bright-field and fluorescence for **(A) **wild-type cells and **(B)** a mutant *S. cerevisiae *strain selected from a global screen for high yielding strains (Bonander et al., 2005). Only the mutant cells produced correctly localized protein.

In the last few years, significant advances have been made in this second approach by understanding how a yeast cell responds to the stress of producing a recombinant protein at a molecular level, and how this information can be used to identify improved host strains (Bonander et al., 2009; Ashe and Bill, 2011; Bawa et al., 2011; Lee et al., 2012). Since *S. cerevisiae* is particularly amenable to studying the mechanistic basis of high-yielding recombinant protein production experiments using the tools of systems and synthetic biology, its more routine use is an obvious way to produce less tractable proteins recombinantly (Drew et al., 2008). Identifying or engineering yeast strains with improved yield characteristics may either be targeted toward one particular pathway or may take a more global approach (Ashe and Bill, 2011). Examples of the targeted approach are provided by the “humanization” of the yeast glycosylation (De Pourcq et al., 2010) and sterol (Kitson et al., 2011) pathways and modifying membrane phospholipid synthesis to proliferate intracellular membranes (Guerfal et al., 2013). Studies taking a more global approach in both *S. cerevisiae* (Bonander et al., 2005; Bonander and Bill, 2009) and *P. pastoris* (Baumann et al., 2011; Rebnegger et al., 2013) have identified the importance of the unfolded protein response (UPR; the cellular stress response activated in response to an accumulation of unfolded or misfolded protein) and reduced translational activity in high yielding cultures. In contrast to the mammalian UPR, the simpler UPR of yeast does not lead to down-regulation of translation to reduce protein synthetic load (Patil and Walter, 2001). We have previously noted that reducing protein synthetic capacity in yeast might be an effective way to improve recombinant protein yields since this capacity is unregulated in response to unfolded protein in cells (Ashe and Bill, 2011). Such insights, which are not yet possible in higher eukaryotic systems, have been used to select specific yeast strains that can substantially improve recombinant yields compared to wild-type cells (Bonander et al., 2009; Norden et al., 2011; **Figure 1**). The minimal use of *S. cerevisiae* as a host shown in **Table 1** is therefore at odds with this unique potential for optimization; it is possible that the increasing popularity of *P. pastoris* has detracted from the use of *S. cerevisiae*. I suggest that this undervalued host system should therefore be revisited, especially in view of its success in the production of challenging targets (Drew et al., 2008).

## YEASTS AS FIRST-CHOICE HOST CELLS IN RECOMBINANT PROTEIN PRODUCTION STRATEGIES

For the majority of researchers, *E. coli* is still the first host cell to be considered in any new protein production experiment; **Table 1** shows it has been consistent in its usage for over 30 years, with the beginnings of a decline in the last 8 years. Large protein production initiatives such as NYSGRC^1^ and OPPF-UK^2^ use *E. coli*, insect, and mammalian cell-lines as routine hosts; yeast is still employed on an *ad hoc* basis and the reasons for that are unclear. Since individual research teams cannot typically afford the time and investment in the full range of available host systems, I propose that a laboratory with the ability to screen for the expression of recombinant genes in *E. coli*, *S. cerevisiae*, and *P. pastoris* would be well placed to produce most target proteins; **Table 1** shows that since 2005, 85–90% of recombinant genes were expressed in these microbes. Data from the Research Collaboratory for Structural Bioinformatics Protein Data Bank (PDB^3^) show that, for soluble proteins in particular, the probability of successful expression in *E. coli* is sufficiently high to justify its premier position in **Table 1** (Ferrer-Miralles et al., 2009). Complementing this, yeasts have the capacity to produce the most challenging proteins: **Figure 1** strikingly demonstrates that the selection of a specific *S. cerevisiae* strain enables this type of bespoke optimization for a eukaryotic membrane protein tagged with green fluorescent protein that could not be produced in *E. coli*. The panels show confocal microscopy images with bright-field and fluorescence for wild-type cells and a mutant *S. cerevisiae* strain selected from a global screen for high yielding strains (Bonander et al., 2005). Only the mutant cells produced correctly localized protein. More broadly, it is notable that for eukaryotic membrane proteins, over half of all the structures deposited in the PDB obtained from recombinant material were from proteins synthesized in *P. pastoris* and *S. cerevisiae* (Bill et al., 2011). This lends further support to the use of these eukaryotic microbes alongside their prokaryotic counterpart for producing the majority of target proteins. Such a strategy also makes sense from a practical perspective, since working with bacteria and yeast require similar techniques, equipment, and approaches. Consequently, both hosts can be used within the same laboratory without the need for additional specialist investment. Yeasts should therefore be considered alongside *E. coli* at an early stage in any serious strategy to produce recombinant proteins.

## Conflict of Interest Statement

The author declares that the research was conducted in the absence of any commercial or financial relationships that could be construed as a potential conflict of interest.

## Footnotes

1 http://www.nysgrc.org/psi3-cgi/index.cgi

2 http://www.oppf.rc-harwell.ac.uk/OPPF/

3 http://www.rcsb.org/pdb/home/home.do
